# Dietary Fiber Intake is Associated with Increased Colonic Mucosal GPR43+ Polymorphonuclear Infiltration in Active Crohn’s Disease

**DOI:** 10.3390/nu7075223

**Published:** 2015-07-01

**Authors:** Mingli Zhao, Weiming Zhu, Jianfeng Gong, Lugen Zuo, Jie Zhao, Jing Sun, Ning Li, Jieshou Li

**Affiliations:** 1 Department of General Surgery, Jinling Hospital, No. 305 East Zhongshan Rd., Nanjing 210002, China; E-Mails: zhao_ming_li@163.com (M.Z.); dr_zhuweiming@126.com (W.Z.); gongjianfeng@aliyun.com (J.G.); zuolugen@126.com (L.Z.); zhj880616@163.com (J.Z.); jingdianjing99@126.com (J.S.); lijieshou@163.com (J.L.); 2 Department of General Surgery, Nanfang Hospital, South Medical University, No. 1838 North Guangzhoudadao Rd., Guangzhou 510515, China

**Keywords:** GPR43/FFAR2, Crohn’s disease, dietary fibre, polymorphonuclear

## Abstract

G protein-coupled receptor 43/free fatty acid receptor 2 (GPR43/FFAR2) is essential for polymorphonuclear (PMN) recruitment. We investigated the expression of GPR43/FFAR2 in the colon from Crohn’s disease patients and whether dietary fiber in enteral nutrition increases GPR43+ polymorphonuclear infiltration in mucosa. Segments of ascending colon and white blood cells from peripheral blood were obtained from 46 Crohn’s disease patients and 10 colon cancer patients. The Crohn’s disease patients were grouped by the activity of disease (active or remission) and enteral nutrition with or without dietary fiber. Histological feature, expression and location of GPR43/FFAR2 and level of tumor necrosis factor-α (TNF-α), interleukine-6 (IL-6) and myeloperoxidase were assessed. The results of hematoxylin-eosin and immunohistochemistry staining revealed that the infiltration of immune cells, including GPR43+ PMN, was more severe in active Crohn’s disease patients who consumed normal food or enteral nutrition with dietary fiber than in remission patients and colon cancer patients. This finding was supported by the results of GPR43 and myeloperoxidase expression. Active Crohn’s disease (CD) patients who consumed enteral nutrition without dietary fiber exhibited severe immune cell infiltration similar to the other active CD patients, but GPR43+ PMNs were rarely observed. The level of TNF-α mRNA in active Crohn’s disease patients was higher than those of the other patients. In conclusion, the use of dietary fiber in enteral nutrition by active Crohn’s disease patients might increase GPR43+ PMNs infiltration in colon mucosa. This effect was not observed in Crohn’s disease patients in remission.

## 1. Introduction

Dietary fibre and short-chain fatty acids (SCFAs) are generally thought to be “good” for colon health. A lack of enteral nutrition, such as occurs during total parenteral nutrition, leads to atrophy and hypofunction throughout the gastrointestinal tract [1], and the addition of dietary fibre could prevent colonic atrophy [2]. Diversion colitis, a non-specific inflammation of a de-functioned segment of intestine after diversion of the fecal stream, could be treated with short-chain fatty acid enemas of the diverted colon [3,4]. Tight junction proteins, such as zonula occludens-1 (ZO-1), occludin and claudin, play crucial roles in intestinal barrier integrity, which seal the paracellular space between epithelial cells, and thus prevent the paracellular diffusion of intestinal bacteria and other antigens across the epithelium. In animal studies, dietary fiber [5] and SCFA [6] have been found to increase the expression of tight junction proteins, such as ZO-1 and occludin. Hallert *et al.* [7] and Fernandez-Banares *et al.* [8] reported the use of dietary fiber in the maintenance of remission in inflammatory bowel disease (IBD) patients. The effect of dietary fiber is usually attributed to an increase in SCFA production in the colonic lumen [9]. SCFAs, especially butyrate, are not only the energy source of colonocytes, but also ligands of G-protein coupled receptor 43.

In 1997, the gene of G-protein coupled receptor 43 (GPR43), along with G-protein coupled receptor 40/41/42 (GPR40/41/42), was found in the course of search for novel subtypes of the unrelated galanin receptor [10]. In 2003, GPR43 and GPR41 were deorphanized by two different teams and were found to be activated by short-chain fatty acids, which is why they are called free fatty acid receptor 2 (FFAR2) and free fatty acid receptor 3 (FFAR3) [11,12]. Researchers also found that GPR43 and GPR41 were coupled with Gq and Gi/o proteins and that their activation induces an increase in intracellular Ca^2^+ and a decrease in intracellular cyclic adenosine monophosphate (cAMP). GPR43/FFAR2 is expressed in adipose tissue, the gastrointestinal tract, and in inflammatory cells (mainly neutrophils). It is believed that GPR43 is involved in lipid metabolism, gastrointestinal tract function, and inflammatory disease [13,14].

Neutrophils (or polymorphonuclears, PMNs) play an important role in the development of Crohn’s disease (CD). It is believed that the intrinsic functions of neutrophils (e.g., phagocytosis, bacterial killing and digestion) in CD are intact [15,16,17], but the recruitment of neutrophils is abnormal. Segal *et al.* [18] and Marks *et al.* [19] found that the number of neutrophils that pass out of a skin window created by dermal abrasion is considerably lower in patients with CD compared with healthy controls. As in these skin windows, a subsequent study found that there is a significant reduction in neutrophil recruitment at trauma sites in rectum and ileum of patients with CD [20]. This reduction might result from a failure of the macrophages to secrete pro-inflammatory cytokines, such as TNF-α, in response to immune activation in CD patients [16], which would result in significantly delayed bacterial clearance [21] and lead to the formation of granulomata [18].

SCFA can recruit immune cells, and this process is mediated by GPR43/FFAR2, which is expressed in neutrophil cells. Neutrophils (or PMNs) from GPR43 knock out (GPR43 KO) mice cannot be recruited by SCFA [22,23,24]. However, the role of GPR43/FFAR2 on colitis is still controversial. Sina *et al.* [23] and Kim *et al.* [25] found that knocking out the GPR43 gene decreases leukocyte infiltration and reduces the severity of colon inflammation in a model of colitis. However, Maslowski *et al.* [22] and Masui *et al.* [26] believe that knocking out the GPR43 increases leukocyte infiltration and increases the severity of colon inflammation in model mice.

Karaki *et al.* [27] investigated the expression of GPR43/FFAR2 in the human colon. Segments of ascending colon were obtained from colon cancer patients undergoing colectomies, and nonpathological regions were cut from the surgical specimens. They found that GPR43 was expressed in enteroendocrine cells containing peptide YY in human colon tissue. We sought to investigate the expression of GPR43/FFAR2 in the colon of CD patients to deduce whether this expression in enteroendocrine cells is the same as that in non-Crohn’s disease patients. We also investigated whether dietary fiber influences the infiltration of GPR43+ immune cells in the mucosa. Zonula occludens 1 (ZO-1, a member of the tight junction proteins) and myeloperoxidase (MPO) were measured as markers of the mechanical barrier [28] and neutrophil infiltration [29].

## 2. Materials and Methods

### 2.1. Patient Population and Ethical Considerations

This was a retrospective study. Ten colon cancer patients (can.) and 46 Crohn’s disease patients were included in our study. Crohn’s disease patients were divided into 5 groups, according to Table 1.

**Table 1:** Groups of the 46 Crohn’s disease patients.

Group	*N* =	Activity of disease	Type of surgery	Enteral nutrition before surgery
ac-food	7	active	emergency	Normal food
ac-EN	10	active	selective	Peptison
ac-DF	9	active	selective	Nutrison
re-EN	10	remission	selective	Peptison
re-DF	10	remission	selective	Nutrison

Two types of enteral nutrition (EN) were used in our study. Peptisorb (Nutricia, Amsterdam, NL) contains no dietary fiber, Nutrison (Nutricia, Amsterdam, NL) contains 7.5 g dietary fiber (DF) for every 500 mL of the preparation, and fruits and vegetables were included in normal food. ac-food: active Crohn’s disease patients comsuming normal food before operation; ac-EN: active Crohn’s disease patients comsuming enteral nutrition without dietary fiber before operation; ac-DF: active Crohn’s disease patients comsuming enteral nutrition with dietary fiber before operation; re-EN: remission Crohn’s disease patients comsuming enteral nutrition without dietary fiber before operation; re-DF: remission Crohn’s disease patients comsuming enteral nutrition with dietary fiber before operation.

The activity of Crohn’s disease was judged by the Crohn’s disease activity index (CDAI) score before operation. Active Crohn’s disease was defined as CDAI ≥ 150, and Crohn’s disease in remission was defined as CDAI < 150. All of the patients underwent an operation during their hospitalization in our hospital from December 2013 to February 2015. For all of the patients with selective operations in our department, enteral nutrition was routinely consumed at least 10 days prior to surgery. Seven patients who received emergency operations without the use of enteral nutrition before surgery were included as controls (ac-food). For all of these included patients, the ascending colon or part of the ascending colon was removed during the operations.

This study was approved by the Ethics Committee of Jinling Hospital (2013NLY-212). All patients signed written consent that their surgical specimen and other information would be used for scientific research.

### 2.2. Preparation of Colon and White Blood Cells

Segments of ascending colon and samples of venous blood were obtained from these 56 patients.

From each patient, 3 segments of the ascending colon (approximately 1 cm × 1 cm) were cut from the region of surgical specimen without visible lesions (can.) but very close to the ulcer (patients of Crohn’s disease). Two segments were frozen at −80 °C, and the third segment was placed in a 4% paraformaldehyde solution.

During hospitalization, 2 mL venous blood was obtained from each patient and anticoagulated by Ethylenediaminetetraacetic acid (EDTA). White blood cells (WBC) were separated and washed by Hank’s buffer twice and then frozen at −80 °C.

### 2.3. Hematoxylin-Eosin Staining and Immunohistochemistry Staining

The process of paraffin embedding, slicing, and Haematoxylin-eosin (H&E) staining followed the standard procedure.

Immunohistochemistry (IHC) staining was used to investigate the location of GPR43 and peptide-YY (PYY). After antigen retrieval (with citrate buffer) and organization background closing (with goat serum), sections of paraffin embedded colon were incubated with primary antibodies (anti-GPR43 (Abcam, Cambridge, UK, 1:200) and anti-PYY (Abcam, Cambridge, UK, 1:100)) for 1 h. Then, the samples were incubated with a biotin-linked second antibody (goat-anti-rabbit) for 20 min, with streptavidin labeled with horseradish-peroxidase (S-A/HRP) for 20 min and with diaminobenzidine (DAB) for 5 min. The sections were then stained with hematoxylin, and the cover slides were fixed with 50% glycerin.

### 2.4. Western Blot

Western blot was used to investigate the amount of ZO-1, GPR43 and Peptide-YY in the mucosa and the amount of GPR43 in the WBC. We chose α-tubulin as the internal control.

The mucosae were separated from frozen human colon samples. The method for the preparation of protein solution from mucosa and WBC followed the standard process.

Equal amounts of protein (20 µL) were separated by electrophoresis in 15% (Peptide-YY, 11 kD), 10% (α-tubulin, 55 kD, GPR43, 37 kD) or 5% (ZO-1, 220 kD) SDS-polyacrylamide gels. Separated protein was transferred onto polyvinylidene difluoride (PVDF) membranes. After being blocked by skimmed milk, the membranes were incubated with primary antibodies (anti-ZO-1 (Cell Signaling Technology (CST), Boston, USA 1:1000), anti-tubulin (Bioworld, Minnesota, USA, 1:5000), anti-GPR43 (Abcam, Cambridge, UK, 1:1000) or anti-PYY (Abcam, Cambridge, UK, 1:1000)) at 4 °C overnight. Then, the PVDF membranes were incubated with the corresponding HRP-linked secondary antibody (goat-anti-rabbit or goat-anti-chick, 1:10,000) for 1 h at room temperature. Standard ECL reagent (for α-tubulin and ZO-1) and Immobilon Western HRP Substrate (Millipore, for GPR43 and PYY) were used for chemiluminescence. Kodak films were used for the detection of fluorescence from the bands of target proteins. The pixel density of the bands on the film was measured using ImageJ software. The data were presented as target protein expression/α-tubulin expression ratios.

### 2.5. Enzyme-Linked Immunosorbent Assay

Enzyme-linked immunosorbent assay (ELISA) was used to measure myeloperoxidase (MPO) in the colon tissue.

The mucosae were separated from frozen human colon samples. Then, they were carefully weighed and homogenized in lysis buffer (200 mM NaCl, 5 mM EDTA, 10 mM Tris, 10% glycine, 1 mM Phenylmethylsulfonyl fluoride (PMSF), 1 mg/mL leupeptide, and 28 mg/mL aprotinin; pH 7.4). Homogenized tissue samples were centrifuged at 1000 × *g* for 15 min at 4 °C. Supernatant was removed and recentrifuged at 1000 × *g* for 15 min. The concentration of myeloperoxidase (MPO) in the supernatant was measured using ELISA kit (Abcam, Cambridge, UK) according to the manufacturer’s protocol. The data were presented as pg/mg tissue.

### 2.6. Quantitative Real-Time Polymerase Chain Reaction Analysis

Quantitative real-time polymerase chain reaction (qPCR) was used to measure the level of interleukine-6 (IL-6) mRNA and tumor necrosis factor-α (TNF-α) mRNA.

The mucosae of frozen human colon samples were separated. The method of extracting mRNA and reverse transcribing into cDNA followed the protocol described in the PrimeScript RT reagent Kit (TaKaRa Bio, Tokyo, Japan). The primers used for our investigation are described in Table 2.

**Table 2:** Primer for polymerase chain reaction.

Primer	Sequence
IL-6 forward	ACTCACCTCTTCAGAACGAATTG
IL-6 reverse	CCATCTTTGGAAGGTTCAGGTTG
TNF-α forward	CCTCTCTCTAATCAGCCCTCTG
TNF-α reverse	GAGGACCTGGGAGTAGATGAG
GAPDH forward	AGGCCGGTGCTGAGTATGTC
GAPDH reverse	TGCCTGCTTCACCACCTTCT

IL-6: interleukine-6; GAPDH: glyceraldehyde-phosphate dehydrogenase.

qPCR was performed using the SYBR Select Master Mix System (Applied Biosystems, Foster City, CA, USA). The mRNA expression level was determined using StepOne Realtime PCR system with a ΔCT relative quantification model. The mRNA of the reference gene, glyceraldehyde-phosphate dehydrogenase (GAPDH) was calculated and used as a normalization factor.

### 2.7. Statistical Analysis

Quantitative data are expressed as the mean ± SD. One-way analysis of variance was used for the calculation of *p*-values. *p*-values < 0.05 were considered statistically significant. Statistical calculations were made using SPSS (Statistical Product and Service Solutions, Chicago, USA) 19.0.

## 3. Results

### 3.1. Clinical Information

The basic clinical information of the patients is presented in Table 3.

**Table 3:** Clinical information about the patients.

Groups	can.	ac-food #	ac-EN	ac-DF	re-EN	re-DF
Female/Male	5/5	2/5	7/3	4/5	4/6	6/4
Age, years *	48.6 ± 13.1 ^a^	34.2 ± 7.2 ^b^	28.4 ± 5.9 ^b^	27.8 ± 5.8 ^b^	35.6 ± 11.1 ^b^	30.0 ± 7.6 ^b^
CRP, mg/L *	-	74.0 ± 19.9 ^a^	38.0 ± 8.9 ^b^	36.2 ± 7.4 ^b^	11.2 ± 3.1 ^c^	11.6 ± 4.9 ^c^
CDAI *	-	365 ± 41 ^a^	237 ± 70 ^b^	215 ± 36 ^b^	113 ± 26 ^c^	106 ± 11 ^c^
Medication history						
Corticosteroid, *n*	-	3	10	9	9	10
5-aminosalicylates, *n*	-	5	10	9	8	9
Immunosuppressive drugs or infliximab, *n*	-	3	6	7	10	9

Notes: CRP: C-reactive protein; CDAI: Crohn’s disease activity index; *: The figures marked with different letters were significantly different, *p* < 0.05; #: Patients in this group (ac-food) received no enteral nutrition because they arrived our hospital with severe complications, and emergency operations were needed, thus there was no chance for the preoperative treatment; *n*, number of patients in every group; can.: colon cancer patients; ac-food: active Crohn’s disease patients comsuming normal food before operation; ac-EN: active Crohn’s disease patients comsuming enteral nutrition without dietary fiber before operation; ac-DF: active Crohn’s disease patients comsuming enteral nutrition with dietary fiber before operation; re-EN: remission Crohn’s disease patients comsuming enteral nutrition without dietary fiber before operation; re-DF: remission Crohn’s disease patients comsuming enteral nutrition with dietary fiber before operation.

### 3.2. Histological Features

**Figure 1:**
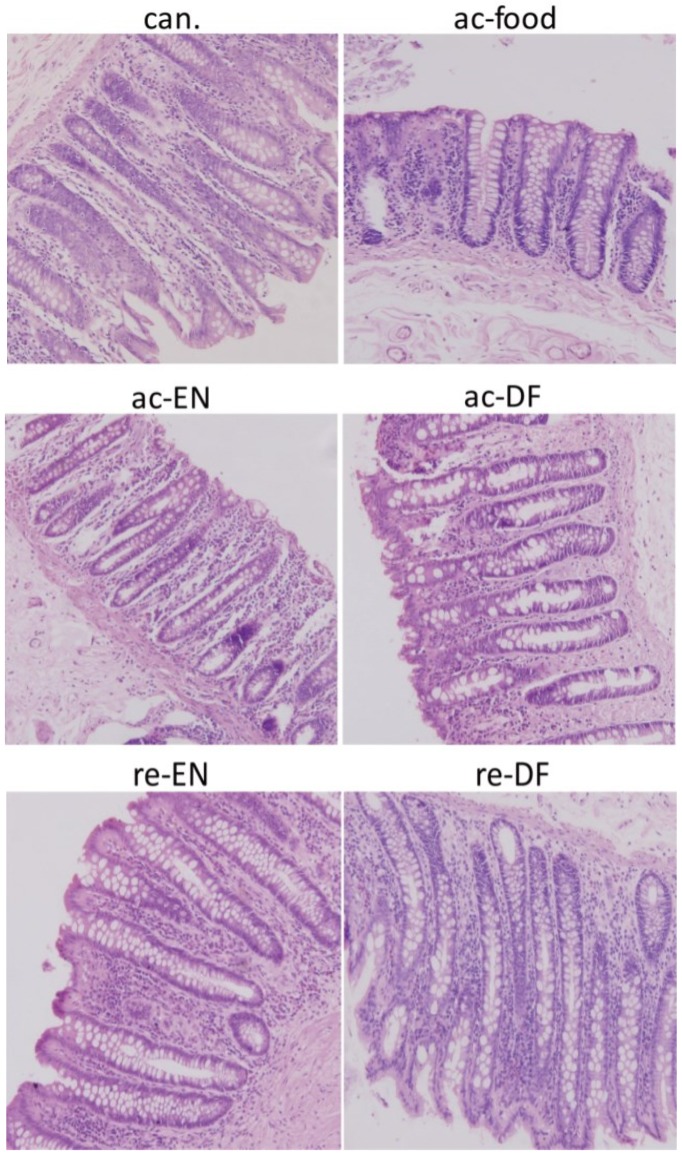
Colon mucosae from different patients. These pictures showed the morphological changes (feature of epithelial and crypt, infiltration of immune cells, and thickness of mucosa) of each group. The most severe morphological damage existed in ac-food group. This damage in re-DF was less severe than that in ac-EN, ac-DF, and re-EN (can.: colon cancer patients; ac-food: active Crohn’s disease patients comsuming normal food before operation; ac-EN: active Crohn’s disease patients comsuming enteral nutrition without dietary fiber before operation; ac-DF: active Crohn’s disease patients comsuming enteral nutrition with dietary fiber before operation; re-EN: remission Crohn’s disease patients comsuming enteral nutrition without dietary fiber before operation; re-DF: remission Crohn’s disease patients comsuming enteral nutrition with dietary fiber before operation.).

Histological features at low magnification (200×, Figure 1) show the morphological changes of mucosae from each group. The morphological feature of mucosae from cancer patients was nearly normal. The most severe morphological damage existed in ac-food group when judged by the feature of epithelial and crypt, infiltration of immune cells and thickness of mucosa. The morphological damage in ac-EN, ac-DF and re-EN was less severe than that in ac-food, but more severe than that in re-DF.

**Figure 2:**
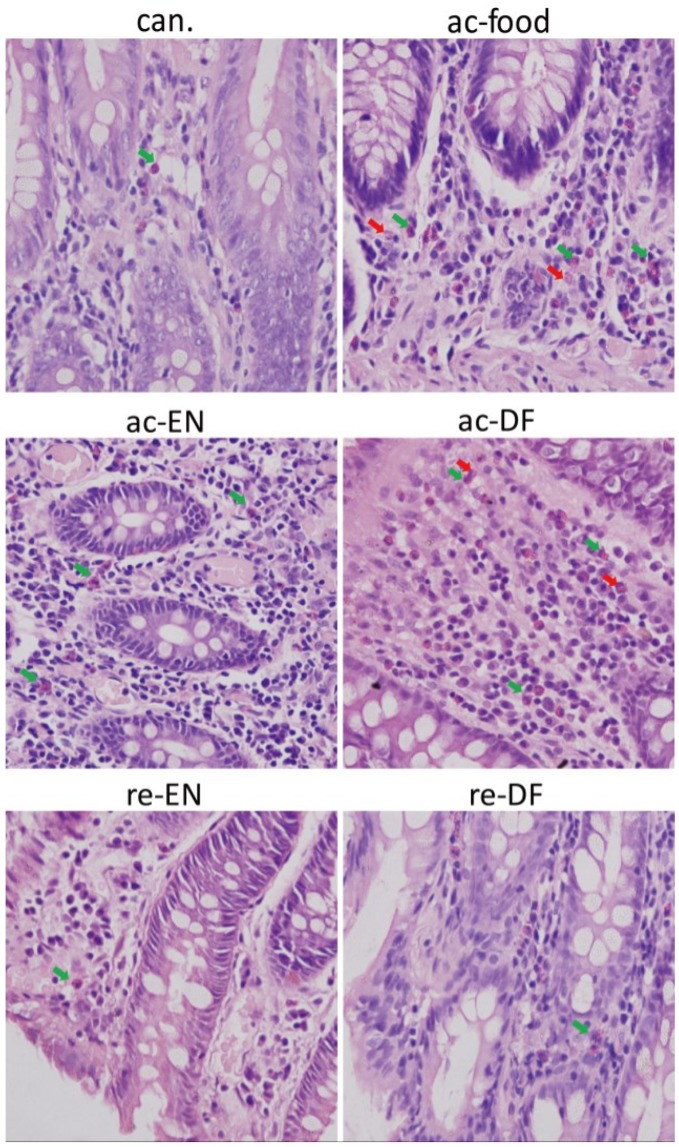
Eosinophils (green arrows) and neutrophils (red arrows) in the mucosae. Mucosae from active Crohn’s disease patients (ac-food, ac-EN and ac-DF) had more immune cells than the others did. Neutrophils could be found in mucosae from active Crohn’s disease patients who had dietary fiber in their food or enteral nutrition (ac-food and ac-DF) (can.: colon cancer patients; ac-food: active Crohn’s disease patients comsuming normal food before operation; ac-EN: active Crohn’s disease patients comsuming enteral nutrition without dietary fiber before operation; ac-DF: active Crohn’s disease patients comsuming enteral nutrition with dietary fiber before operation; re-EN: remission Crohn’s disease patients comsuming enteral nutrition without dietary fiber before operation; re-DF: remission Crohn’s disease patients comsuming enteral nutrition with dietary fiber before operation.).

At high magnification (600×, Figure 2), we found more immune cells in the mucosa from the active Crohn’s disease patients (ac-food, ac-EN and ac-DF). The mucosae from the active Crohn’s disease patients (ac-food, ac-EN and ac-DF) showed more eosinophils (green arrows) and lymphocytes than those of the Crohn’s disease patients in remission (re-EN and re-DF) and the non-Crohn’s disease patients (con. and can.). Neutrophils (red arrows) were found in the mucosae from the active Crohn’s disease patients who used dietary fiber in their food (ac-food) or in enteral nutrition (ac-DF). The total number of infiltrated immune cells showed no obvious difference between the Crohn’s disease patients that used enteral nutrition with or without dietary fiber (ac-EN *vs.* ac-DF; re-EN *vs.* re-DF).

### 3.3. Cytokines

**Figure 3:**
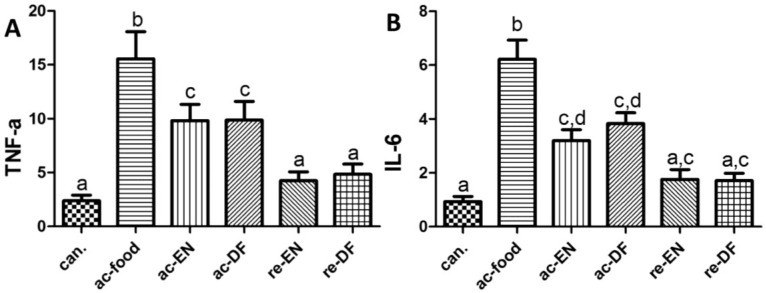
The mRNA of tumor necrosis factor-α (TNF-α) (**A**) (can. 2.40 ± 1.15, ac-food 15.56 ± 5.60, ac-EN 9.83 ± 3.34, ac-DF 9.87 ± 3.87, re-EN 4.25 ± 1.86, and re-DF 4.84 ± 2.12) and IL-6 (**B**) (can. 0.94 ± 0.42, ac-food 6.22 ± 1.59, ac-EN 3.19 ± 0.91, ac-DF 3.83 ± 0.89, re-EN 1.76 ± 0.82, and re-DF 1.71 ± 0.61) in mucosae from different groups. Columns with different letters are significantly different (*p* < 0.05). TNF-α in active Crohn’s patients (ac-food, ac-EN and ac-DF) was higher than that of the Crohn’s disease patients in remission (re-EN and re-DF) and colon cancer patients (can.). The IL-6 of the severe Crohn’s disease patients (ac-food) was higher than in the other patients(can.: colon cancer patients; ac-food: active Crohn’s disease patients comsuming normal food before operation; ac-EN: active Crohn’s disease patients comsuming enteral nutrition without dietary fiber before operation; ac-DF: active Crohn’s disease patients comsuming enteral nutrition with dietary fiber before operation; re-EN: remission Crohn’s disease patients comsuming enteral nutrition without dietary fiber before operation; re-DF: remission Crohn’s disease patients comsuming enteral nutrition with dietary fiber before operation.).

We used qPCR to compare TNF-α mRNA and IL-6 mRNA in different groups. GAPDH mRNA was used as the normalization factor. As shown in Figure 3, ac-food showed the highest expression of TNF-α and IL-6, which might be related to the severity of colon inflammation. The active Crohn’s disease patients (ac-food, ac-EN and ac-DF) showed a significantly higher level of TNF-α and a non-significantly higher level of IL-6 than that of the Crohn’s disease patients in remission (re-EN and re-DF) and colon cancer patients (can.). No significant difference between the Crohn’s disease patients who use enteral nutrition with or without dietary fiber (ac-EN *vs.* ac-DF; re-EN *vs.* re-DF) could be found.

### 3.4. Expression and Location of GPR43

**Figure 4:**
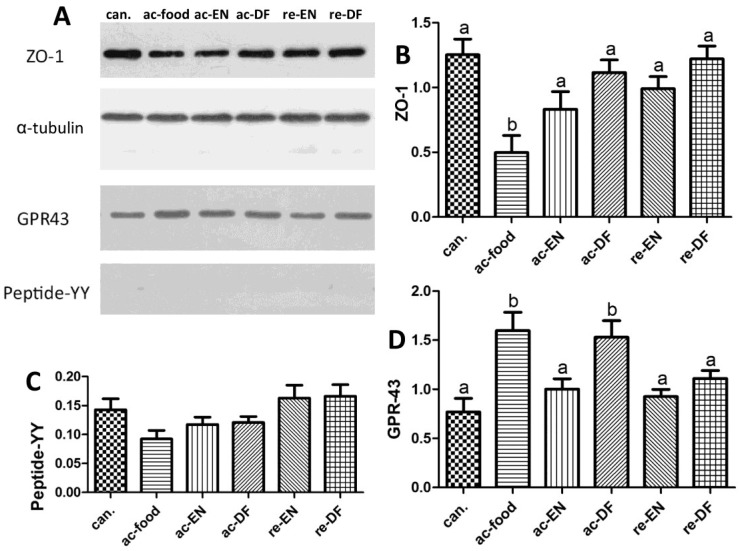
the amount of zonula occludens-1 (ZO-1), G protein-coupled receptor 43 (GPR43) and Peptide-YY in the mucosa and the amount of GPR43 in the White blood cells (WBC). The expression of ZO-1 (can. 1.25 ± 0.27, ac-food 0.50 ± 0.29, ac-EN 0.83 ± 0.30, ac-DF 1.12 ± 0.22, re-EN 0.99 ± 0.21, and re-DF 1.22 ± 0.22), GPR43 (can. 0.77 ± 0.31, ac-food 1.60 ± 0.42, ac-EN 1.00 ± 0.24, ac-DF 1.53 ± 0.38, re-EN 0.93 ± 0.16, and re-DF 1.11 ± 0.18) and peptide-YY (can. 0.29 ± 0.09, ac-food 0.19 ± 0.07, ac-EN 0.23 ± 0.06, ac-DF 0.24 ± 0.05, re-EN 0.33 ± 0.10, and re-DF 0.33 ± 0.09) in colonic mucosae from different groups. Columns with different letters are significantly different (**B**, **D**) (*p* < 0.05). ZO-1 in ac-food was significantly lower than in the others. GPR-43 in ac-food and ac-DF was significantly higher than that in the others. No significant difference of peptide-YY was found among these groups (**C**). (ZO-1: zonula occludens-1; GPR-43: G-protein coupled receptor 43. See notes of Table 1 for the definition of each group; can.: colon cancer patients; ac-food: active Crohn’s disease patients comsuming normal food before operation; ac-EN: active Crohn’s disease patients comsuming enteral nutrition without dietary fiber before operation; ac-DF: active Crohn’s disease patients comsuming enteral nutrition with dietary fiber before operation; re-EN: remission Crohn’s disease patients comsuming enteral nutrition without dietary fiber before operation; re-DF: remission Crohn’s disease patients comsuming enteral nutrition with dietary fiber before operation.).

The result of Western blot (Figure 4) showed that the expression of ZO-1 (Figure 4B) in the ac-food group was significantly lower than that in the other groups, which might be related to the more severe morphological change in the ac-food patients (Table 3). We also found that the ZO-1 in the Crohn’s disease patients who used enteral nutrition with dietary fiber (ac-DF and re-DF) was higher than in the Crohn’s disease patients who used enteral nutrition without dietary fiber (ac-EN and re-EN) but was also not significant. The colonic mucosae from the active Crohn’s disease patients who had dietary fiber in their food or enteral nutrition (ac-DF and re-DF) expressed more GPR43 than those from the other patients (Figure 4D), and this difference was significant. The expression of peptide-YY (Figure 4C) had no significant differences among these seven groups.

**Figure 5:**
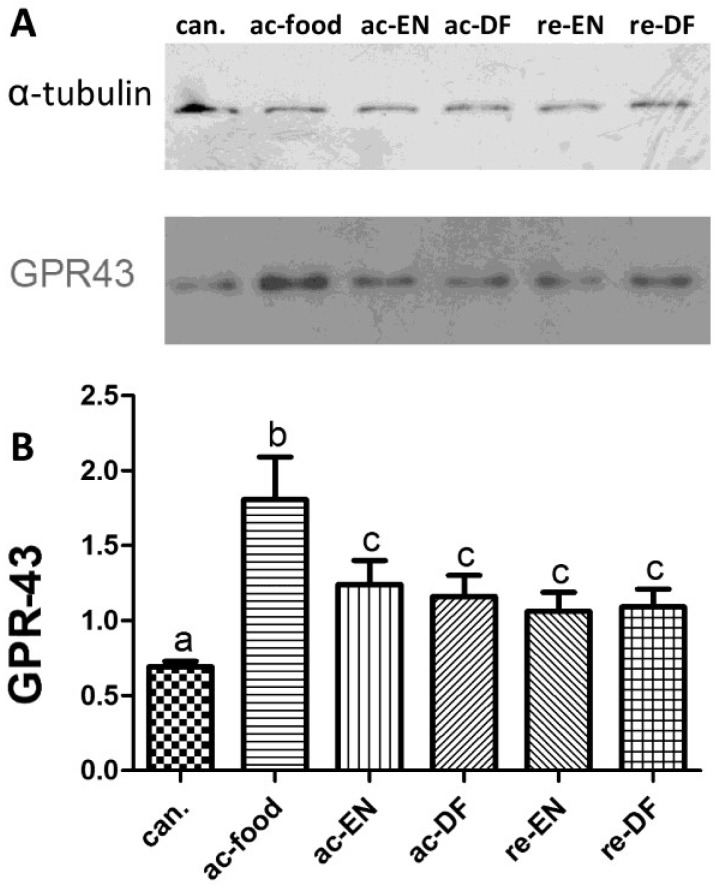
The expression of G-protein coupled receptor 43 (GPR43) in white blood cells from peripheral blood (can. 0.69 ± 0.08, ac-food 1.81 ± 0.40, ac-EN 1.24 ± 0.18, ac-DF 1.16 ± 0.20, re-EN 1.06 ± 0.28, and re-DF 1.09 ± 0.27). Columns with different letters are significantly different (a *vs.* c *p* < 0.05, b *vs.* a and c *p* < 0.01). GPR43 from white blood cell (WBC) of ac-food is much higher than that in the other groups, (can.: colon cancer patients; ac-food: active Crohn’s disease patients comsuming normal food before operation; ac-EN: active Crohn’s disease patients comsuming enteral nutrition without dietary fiber before operation; ac-DF: active Crohn’s disease patients comsuming enteral nutrition with dietary fiber before operation; re-EN: remission Crohn’s disease patients comsuming enteral nutrition without dietary fiber before operation; re-DF: remission Crohn’s disease patients comsuming enteral nutrition with dietary fiber before operation.).

The expression of GPR43 in WBC from peripheral blood is shown in Figure 5. The GPR43 from the WBC of the ac-food group was much higher than that of the other groups. The GPR43 expressed in the other Crohn’s disease patients (ac-EN, ac-DF, re-EN and re-DF) was significantly higher than that in the patients with colon cancer (can.). No difference could be found between the Crohn’s disease patients who used enteral nutrition with or without dietary fiber (ac-EN *vs.* ac-DF; re-EN *vs.* re-DF).

**Figure 6:**
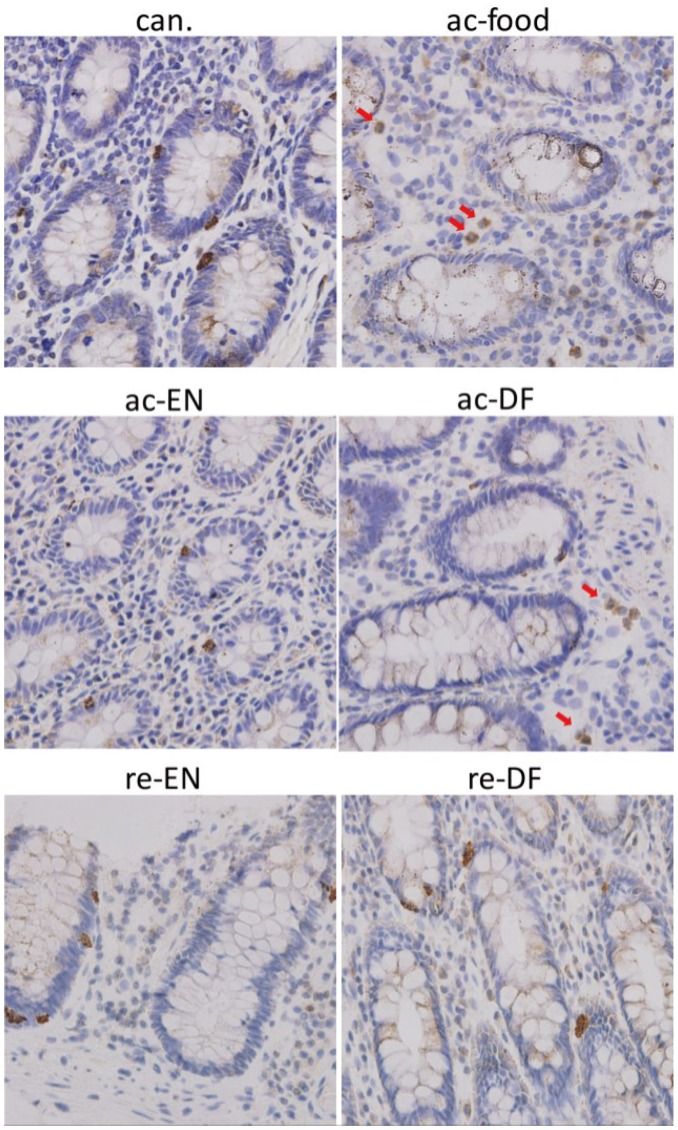
Immunohistochemistry showed the expression of G-protein coupled receptor 43 (GPR43) in crypt and lamina propria. For all these groups, GPR43 high expressing epithelial cells could be found in the crypt. GPR43 high expressing cells could be found in lamina propria in ac-food and ac-DF. These GPR43 high expressing cells in lamina propria had small granules in the cytoplasm and segmented nucleus, which illustrated that they might be PMNs (polymorphonuclears). (can.: colon cancer patients; ac-food: active Crohn’s disease patients comsuming normal food before operation; ac-EN: active Crohn’s disease patients comsuming enteral nutrition without dietary fiber before operation; ac-DF: active Crohn’s disease patients comsuming enteral nutrition with dietary fiber before operation; re-EN: remission Crohn’s disease patients comsuming enteral nutrition without dietary fiber before operation; re-DF: remission Crohn’s disease patients comsuming enteral nutrition with dietary fiber before operation.).

**Figure 7:**
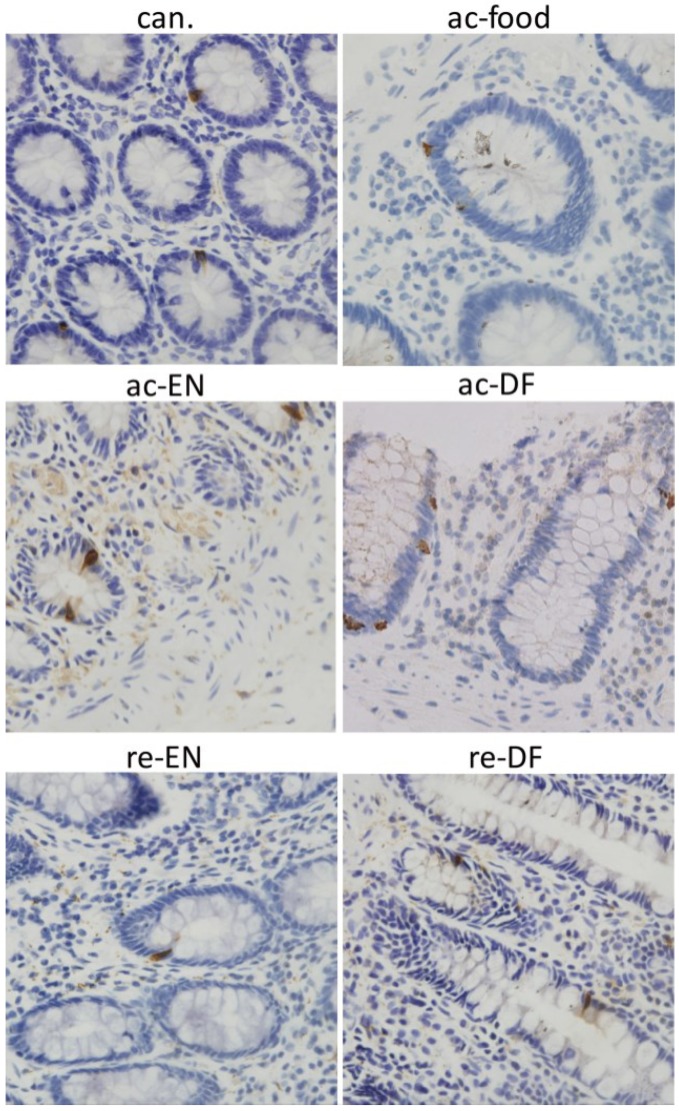
Expression of peptide-YY in crypt and lamina propria. For all these groups, peptide-YY high expressing cells could be found in the crypt. Peptide-YY+ cells could not be found in lamina proprial cells in any groups, (can.: colon cancer patients; ac-food: active Crohn’s disease patients comsuming normal food before operation; ac-EN: active Crohn’s disease patients comsuming enteral nutrition without dietary fiber before operation; ac-DF: active Crohn’s disease patients comsuming enteral nutrition with dietary fiber before operation; re-EN: remission Crohn’s disease patients comsuming enteral nutrition without dietary fiber before operation; re-DF: remission Crohn’s disease patients comsuming enteral nutrition with dietary fiber before operation.).

The location of GPR43 was detected with immunohistochemistry. For all of these groups, GPR43 immunoreactivity could be found in colon epithelial cells and was especially high in some crypt cells. Dotted staining could be found in all the epithelial cells. GPR43 high expressing epithelial cells were found in the crypt (Figure 6). In addition to in the colon epithelium, GPR43 high expressing cells could be found in lamina proprial cells in the colon from only the active Crohn’s disease patients who had dietary fiber in their food or received enteral nutrition with dietary fiber (ac-food and ac-DF) (Figure 6). The shape of these cells and nucleus illustrates that these GPR43 high expressing cells in lamina propria were immune cells that were most likely PMN. Peptide-YY immunoreactivity could be found in some epithelial cells in the crypt in colon of all the groups (Figure 7). However, unlike GPR43, peptide-YY+ cells could not be found in lamina propria cells in any group.

### 3.5. Expression of MPO

**Figure 8:**
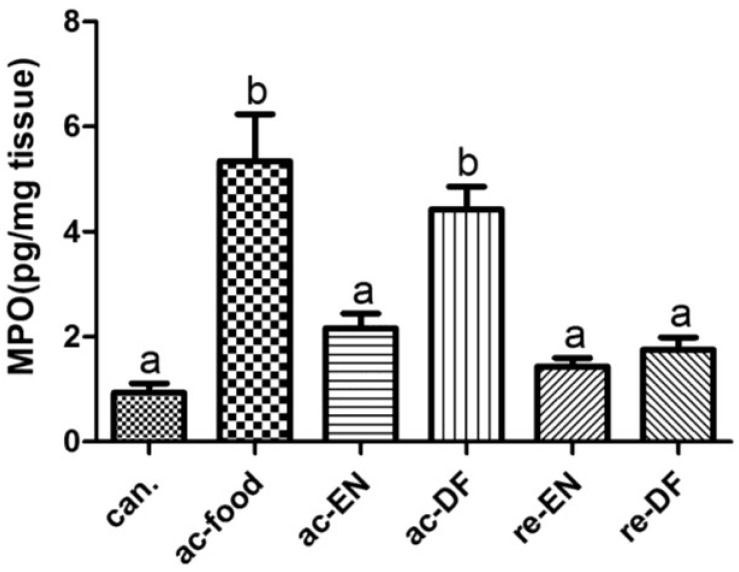
The expression of myeloperoxidase (MPO) in colon mucosae from different groups (pg/mg tissue) (can. 0.94 ± 0.38, ac-food 5.34 ± 2.00, ac-EN 2.16 ± 0.64, ac-DF 4.43 ± 0.95, re-EN 1.44 ± 0.35, and re-DF 1.76 ± 0.51). Columns with different letters are significantly different (*p* < 0.01). MPO in colon mucosa from ac-food and ac-DF was significantly higher than that in the other groups, (can.: colon cancer patients; ac-food: active Crohn’s disease patients comsuming normal food before operation; ac-EN: active Crohn’s disease patients comsuming enteral nutrition without dietary fiber before operation; ac-DF: active Crohn’s disease patients comsuming enteral nutrition with dietary fiber before operation; re-EN: remission Crohn’s disease patients comsuming enteral nutrition without dietary fiber before operation; re-DF: remission Crohn’s disease patients comsuming enteral nutrition with dietary fiber before operation.).

The expression of myeloperoxidase (MPO) in colon mucosa was measured using ELISA. The result of ELISA (Figure 8) revealed that the expression of MPO in colonic mucosae from the active Crohn’s disease patients who had dietary fiber in their food or enteral nutrition (ac-DF and re-DF) was significantly higher than that in the other groups.

## 4. Discussion

Our study observed the histological feature and expression of ZO-1 in the colon of Crohn’s disease patients. ZO-1 is a tight junction protein and can be used as a marker of gut mechanical barrier [28,30]. Combining the histological feature and expression of ZO-1 detected with Western blot, we found that the patients with severe Crohn’s disease (ac-food) exhibited the most impaired mucosa, which was significantly different from those of the other groups. This finding suggests that Crohn’s disease can impair colon mucosa, which is widely accepted and regarded as a diagnostic point of IBD [31]. The Crohn’s disease patients who used enteral nutrition (ac-EN, ac-DF, re-EN and re-DF) showed no significant difference from colon cancer patients (can.). This indicates that enteral nutrition can repair mucosal damage [32,33].

GPR43/FFAR2 is believed to be expressed in adipose tissue, enteroendocrine cells and immune cells (particularly in neutrophils) [13,14]. In our study, the expression and localization of GPR43/FFAR2 in colon was investigated with Western blot and IHC staining. We found GPR43 high expressing epithelial cells in the crypt, and the distribution of these cells was similar to the distribution of peptide-YY+ cells, which indicates that these GPR43 high expressing and peptide-YY+ cells in the crypt are enteroendocrine cells [27]. In addition to the crypt, GPR43+ cells could also be found in lamina propria but only in the colon of ac-food and ac-DF patients (active Crohn’s disease patients who had dietary fiber in their food or received enteral nutrition). Judging by the small granules in cytoplasm and the segmented nucleus, these GPR43+ cells in lamina propria might be PMN. If we combined the H&E staining at high magnification with the IHC staining, we could further determine that these GPR43+ cells in lamina propria should be PMNs. We compared the expression of GPR43 and peptide-YY among different groups using Western blot. The expression GPR43 in the colon mucosae of ac-food and ac-DF was significantly higher than that in the other groups, and the expression of peptide-YY showed no difference among these groups. We could conclude that the differential expression of GPR43 resulted from the difference of GPR43+ PMN infiltration rather than the difference of enteroendocrine cells, which express both GPR43 and peptide-YY. MPO is typically assessed as a marker of neutrophils infiltration and accumulation into the inflamed colon tissue [29]. The MPO ELISA results also indicated that there was more severe neutrophils infiltration in the mucosae of ac-food and ac-DF groups than that in the other groups.

In our study, GPR43 in WBC from peripheral blood was also detected by Western blot. We found that GPR43 in WBC from the ac-food group was significantly higher than that in the other groups, and this figure (Figure 5B) was similar to that of TNF-α and IL-6 (Figure 3). Both TNF-α and IL-6 are usually used to reflect the severity of inflammation, which indicates that the GPR43 in WBC may be associated with the severity of inflammation.

The health benefits of dietary fiber have long been appreciated. Dietary fibers that can influence gastrointestinal microflora are termed prebiotics [34]. These fibers can be fermented into short-chain fatty acids [35] and regulate gastrointestinal inflammation [36]. The receptor of short-chain fatty acid, GPR43, is an important link between dietary fiber and its influence on inflammation. GPR43 particularly highly expressed in PMNs. At least three *in vitro* studies have confirmed that FFAR2/GPR43 is essential for PMN migration induced by SCFAs, and PMN from GPR43 KO mice cannot respond to SCFA [22,23,24]. This finding made GPR43 a potential target for the treatment of colitis. However, *in vivo* studies of GPR43 KO mice made this foreground complex. In one such study, GPR43 KO mice showed more severe inflammation and increased PMN infiltration/activation in the model of dextran sulfate sodium (DSS)-induced colitis [22]. GPR43 KO mice also showed reduced colon length and increased daily activity index (DAI). In another study, GPR43 KO mice exhibited thicker mucosa, greater infiltration of inflammatory cells, and a higher level of crypt damage compared with the colitis model of wild-type mice. The colitis can be ameliorated by SCFA in wild-type mice but not in GPR43 KO mice [26]. These studies suggest that a GPR43 agonist should be used for the treatment of colitis. However, other studies reported different results. One study used the same model of colitis, but histological examination of the colon showed reduced tissue damage and significantly reduced PMN in the submucosal tissue in GPR43 KO mice compared with that of wild-type mice. Additionally, the level of TNF-α in GPR43 KO mice was significantly reduced [23]. In the model of 2, 4, 6-trinitrobenzene sulfonic-acid (TNBS) induced colitis, the GPR43 KO mice revealed milder decreases of colon length and less weight loss. Histological examination revealed milder leukocyte infiltration, mucosa hyperplasia, and tissue damage in GPR43 KO mice than in the wild-type mice [25]. These studies suggest that the GPR43 antagonist but not the agonist should be used. This contradiction puzzled us: A link between GPR43/FFAR2 and IBD has been established, but it is unclear whether the agonist or antagonist of FFA2 would be the preferred treatment [14]. In our study, recruitment of neutrophils induced by dietary fiber in enteral nutrition occurred in only patients with active Crohn’s disease (ac-DF) but not in patients in remission (re-DF). This finding indicate that enteral nutrition with dietary fiber could not increase the neutrophil infiltration of colon mucosa in patients with Crohn’s disease in remission, which might be explained by the fact that TNF-α increases tight-junction permeability and promotes the chemotaxis induced by SCFA [37,38]. Although there was more severe GPR43+ PMNs infiltration in ac-DF than that in ac-EN, the ac-DF patients exhibited neither lower ZO-1 nor higher cytokine than the ac-EN group. This finding might be explained if dietary fiber and SCFA have some other effects on colon barrier and inflammation through pathway that does not involve GPR43 and the recruitment of immune cells [36,39,40]. Dietary fiber might increase neutrophil recruitment, but does it benefit CD patients?

There is a perspective that Crohn’s disease is a consequence of innate immune deficiency [16,41], and the deficiency in neutrophil recruitment plays an important role in the development of Crohn’s disease lesions [42]. Approximately 50% of patients with chronic granulomatous disease (which results from inherited primary immune deficiencies in neutrophil function) develop non-infectious chronic bowel inflammation that bears a striking resemblance to CD in clinical, endoscopic, and histopathological assessment [43]. Compared with healthy controls or patients with rheumatoid arthritis, the numbers of neutrophils that pass out of the skin windows created by dermal abrasion are lower in patients with Crohn’s disease [18,19]. This finding might be due to a deficient local inflammatory response [44]. In another study [20], endoscopic pinch biopsies were taken from non-inflamed mucosae to induce trauma in the rectum and ileum of CD patients. Neutrophil recruitment was observed 6 h later. As in the skin windows, there was a significant reduction in neutrophil recruitment in patients with CD. This reduction was not observed in patients with Ulcerative Colitis (UC). This finding might be due to the failure of macrophages to secrete pro-inflammatory cytokines such as TNF-α in response to immune activation in CD patients [16]. This failure would result in significantly delayed bacterial clearance [21] and lead to the formation of granulomata [18]. Sewell *et al.* [45] proposed a three-stage model to explain the formation of granulomata in CD patients. The first stage of this model involves the penetration of the bowel wall by luminal contents facilitated by environmental factors (e.g., infection) or defects of the mucosal barrier. In the second stage, macrophages fail to secrete pro-inflammatory cytokines, particularly TNF-α, in sufficient quantities to adequately trigger an acute inflammatory response, which results in a reduced influx of neutrophils. Finally, as a consequence of the impaired neutrophil recruitment, the bacteria persist within the tissue and are phagocytosed by macrophages, which form granulomata in an attempt to contain the bacteria. In our study, dietary fiber in enteral nutrition increased GPR43+ neutrophil infiltration. This increase might enhance the impaired neutrophil recruitment in CD patients and improve the bacterial clearance, and this supposition would provide a new explanation of the benefit of dietary fiber to CD patients.

There are some limitations to our study. Our study was retrospective, and only 7~10 patients were included in each group. Two types of enteral nutrition (Peptison and Nutrison) were consumed by the patients, but the presence of dietary fiber was not the only difference between these two types of enteral nutrition (Peptison contained polypeptide, and Nutrison contained total protein). Therefore, the difference between ac-DF and ac-EN could not be attributed solely to dietary fiber. We did not measure the SCFA in the colon or the permeability of the epithelial cells directly. Additional prospective studies are needed to reveal the true interactions of dietary fiber/SCFA with GPR43 in colitis.

## 5. Conclusions

In conclusion, dietary fiber in enteral nutrition used by patients with active Crohn’s disease increased PMN infiltration in lamina propria. This effect was not observed in Crohn’s disease in remission. Further research is needed to evaluate whether it is useful for CD patients to improve the impaired neutrophil recruitment.

## References

[1] Ford W.D., Boelhouwer R.U., King W.W., de Vries J.E., Ross J.S., Malt R.A. (1984). Total parenteral nutrition inhibits intestinal adaptive hyperplasia in young rats: Reversal by feeding. Surgery.

[2] Jacobs L.R., Lupton J.R. (1984). Effect of dietary fibers on rat large bowel mucosal growth and cell proliferation. Am. J. Physiol..

[3] Harig J.M., Soergel K.H., Komorowski R.A., Wood C.M. (1989). Treatment of diversion colitis with short-chain-fatty acid irrigation. N. Engl. J. Med..

[4] Komorowski R.A. (1990). Histologic spectrum of diversion colitis. Am. J. Surg. Pathol..

[5] Chen H., Mao X., He J., Yu B., Huang Z., Yu J., Zheng P., Chen D. (2013). Dietary fibre affects intestinal mucosal barrier function and regulates intestinal bacteria in weaning piglets. Br. J. Nutr..

[6] Ma X., Fan P.X., Li L.S., Qiao S.Y., Zhang G.L., Li D.F. (2012). Butyrate promotes the recovering of intestinal wound healing through its positive effect on the tight junctions. J. Anim. Sci..

[7] Hallert C., Kaldma M., Petersson B.G. (1991). Ispaghula husk may relieve gastrointestinal symptoms in ulcerative colitis in remission. Scand. J. Gastroenterol..

[8] Fernandez-Banares F., Hinojosa J., Sanchez-Lombrana J.L., Navarro E., Martínez-Salmerón J.F., García-Pugés A., González-Huix F., Riera J., González-Lara V., Domínguez-Abascal F. (1999). Randomized clinical trial of Plantago ovata seeds (dietary fiber) as compared with mesalamine in maintaining remission in ulcerative colitis. Am. J. Gastroenterol..

[9] Galvez J., Rodriguez-Cabezas M.E., Zarzuelo A. (2005). Effects of dietary fiber on inflammatory bowel disease. Mol. Nutr. Food Res..

[10] Sawzdargo M., George S.R., Nguyen T., Xu S., Kolakowski L.F., O’Dowd B.F. (1997). A cluster of four novel human G protein-coupled receptor genes occurring in close proximity to CD22 gene onchromosome19q13.1. Biochem. Biophys. Res. Commun..

[11] Brown A.J., Goldsworthy S.M., Barnes A.A., Eilert M.M., Tcheang L., Daniels D., Muir A.I., Wigglesworth M.J., Kinghorn I., Fraser N.J. (2003). The orphan G protein-coupled receptors GPR41 and GPR43 are activated by propionate and other short chain carboxylic acids. J. Biol. Chem..

[12] Le Poul E., Loison C., Struyf S., Springael J.Y., Lannoy V., Decobecq M.E., Brezillon S., Dupriez V., Vassart G., Van Damme J. (2003). Functional characterization of human receptors for short chain fatty acids and their role in polymorphonuclear cell activation. J. Biol. Chem..

[13] Bindels L.B., Dewulf E.M., Delzenne N.M. (2013). GPR43/FFA2: Physiopathological relevance and therapeutic prospects. Trends Pharmacol. Sci..

[14] Ulven T. (2012). Short-chain free fatty acid receptors FFA2/GPR43 and FFA3/GPR41 as new potential therapeutic targets. Front. Endocrinol. (Lausanne).

[15] Hermanowicz A., Gibson P.R., Jewell D.P. (1985). The role of phagocytes in inflammatory bowel disease. Clin. Sci. (Lond.).

[16] Hayee B., Rahman F.Z., Sewell G., Smith A.M., Segal A.W. (2010). Crohn’s disease as an immunodeficiency. Expert Rev. Clin. Immunol..

[17] Hayee B.H., Rahman F.Z., Tempero J., McCartney S., Bloom S.L., Segal A.W., Smith A.M. (2011). The neutrophil respiratory burst and bacterial digestion in Crohn’s disease. Dig. Dis. Sci..

[18] Segal A.W., Loewi G. (1976). Neutrophil dysfunction in Crohn’s disease. Lancet.

[19] Marks D.J.B., Radulovic M., McCartney S., Bloom S., Segal A.W. (2007). Modified skin window technique for the extended characterisation of acute inflammation in humans. Inflamm. Res..

[20] Marks D.J.B., Harbord M.W.N., MacAllister R., Rahman F.Z., Young J., Al-Lazikani B., Lees W., Novelli M., Bloom S., Segal A.W. (2006). Defective acute inflammation in Crohn’s disease: A clinical investigation. Lancet.

[21] Smith A.M., Rahman F.Z., Hayee B.H., Graham S.J., Marks D.J., Sewell G.W., Palmer C.D., Wilde J., Foxwell B.M., Gloger I.S. (2009). Disordered macrophage cytokine secretion underlies impaired acute inflammation and bacterial clearance in Crohn’s disease. J. Exp. Med..

[22] Maslowski K.M., Vieira A.T., Ng A., Kranich J., Sierro F., Yu D., Schilter H.C., Rolph M.S., Mackay F., Artis D. (2009). Regulation of inflammatory responses by gut microbiota and chemoattractant receptor GPR43. Nature.

[23] Sina C., Gavrilova O., Forster M., Till A., Derer S., Hildebrand F., Raabe B., Chalaris A., Scheller J., Rehmann A. (2009). G protein-coupled receptor 43 is essential for neutrophil recruitment during intestinal inflammation. J. Immunol..

[24] Vinolo M.A., Ferguson G.J., Kulkarni S., Damoulakis G., Anderson K., Bohlooly-Y M., Stephens L., Hawkins P.T., Curi R. (2011). SCFAs induce mouse neutrophil chemotaxis through the GPR43 receptor. PLoS ONE.

[25] Kim M.H., Kang S.G., Park J.H., Yanagisawa M., Kim C.H. (2013). Short-chain fatty acids activate GPR41 and GPR43 on intestinal epithelial cells to promote inflammatory responses in mice. Gastroenterology.

[26] Masui R., Sasaki M., Funaki Y., Ogasawara N., Mizuno M., Iida A., Ogasawara N., Mizuno M., Iida A., Izawa S. (2013). G protein-coupled receptor 43 moderates gut inflammation through cytokine regulation from mononuclear cells. Inflamm. Bowel Dis..

[27] Karaki S., Tazoe H., Hayashi H., Kashiwabara H., Tooyama K., Suzuki Y., Kuwahara A. (2008). Expression of the short-chain fatty acid receptor, GPR43, in the human colon. J. Mol. Histol..

[28] Peng L., Li Z.R., Green R.S., Holzman I.R., Lin J. (2009). Butyrate enhances the intestinal barrier by facilitating tight junction assembly via activation of AMP-activated protein kinase in Caco-2 cell monolayers. J. Nutr..

[29] Daniel C., Sartory N., Zahn N., Geisslinger G., Radeke H.H., Stein J.M. (2007). FTY720 ameliorates Th1-mediated colitis in mice by directly affecting the functional activity of CD4 + CD25 + regulatory T cells. J. Immunol..

[30] Dai C., Zhao D.H., Jiang M. (2012). VSL#3 probiotics regulate the intestinal epithelial barrier *in vivo* and *in vitro* via the p38 and ERK signaling pathways. Int. J. Mol. Med..

[31] Jenkins D., Balsitis M., Gallivan S., Dixon M.F., Gilmour H.M., Shepherd N.A., Theodossi A., Williams G.T. (1997). Guidelines for the initial biopsy diagnosis of suspected chronic idiopathic inflammatory bowel disease. J. Clin. Pathol..

[32] Fell J.M., Paintin M., Arnaud-Battandier F., Beattie R.M., Hollis A., Kitching P., Donnet-Hughes A., MacDonald T.T., Walker-Smith J.A. (2000). Mucosal healing and a fall in mucosal pro-inflammatory cytokine mRNA induced by a specific oral polymeric diet in paediatric Crohn’s disease. Aliment. Pharmacol. Ther..

[33] Borrelli O., Cordischi L., Cirulli M., Paganelli M., Labalestra V., Uccini S., Russo P.M., Cucchiara S. (2006). Polymeric diet alone *versus* corticosteroids in the treatment of active pediatric Crohn’s disease: A randomized controlled open-label trial. Clin. Gastroenterol. Hepatol..

[34] Slavin J. (2013). Fiber and prebiotics: Mechanisms and health benefits. Nutrients.

[35] Reimer R.A., Maathuis A.J., Venema K., Michael R.L., Roland J.G., Wood S. (2014). Effect of the novel polysaccharide PolyGlycopleX® on short-chain fatty acid production in a computer-controlled *in vitro* model of the human large intestine. Nutrients.

[36] Vinolo M.A., Rodrigues H.G., Nachbar R.T., Curi R. (2011). Regulation of inflammation by short chain fatty acids. Nutrients.

[37] Marini M., Bamias G., Rivera-Nieves J., Moskaluk C.A., Hoang S.B., Ross W.G., Pizarro T.T., Cominelli F. (2003). TNF-α neutralization ameliorates the severity of murine Crohn’s-like ileitis by abrogation of intestinal epithelial cell apoptosis. Proc. Natl. Acad. Sci. USA.

[38] Su L., Nalle S.C., Shen L., Singh G., Breskin L.A., Khramtsova E.A., Khramtsova G., Tsai P.Y., Fu Y.X., Abraham C. (2013). TNFR2 activates MLCK-dependent tight junction dysregulation to cause apoptosis-mediated barrier loss and experimental colitis. Gastroenterology.

[39] Nasef N.A., Mehta S., Murray P., Marlow G., Ferguson L.R. (2014). Anti-inflammatory activity of fruit fractions *in vitro*, mediated through toll-like receptor 4 and 2 in the context of inflammatory bowel disease. Nutrients.

[40] Gomez-Hurtado I., Santacruz A., Peiro G., Zapater P., Gutiérrez A., Pérez-Mateo M., Sanz Y., Francés R. (2011). Gut microbiota dysbiosis is associated with inflammation and bacterial translocation in mice with CCl4-induced fibrosis. PLoS ONE.

[41] Vinh D.C., Behr M.A. (2013). Crohn’s as an immune deficiency: From apparent paradox to evolving paradigm. Expert Rev. Clin. Immunol..

[42] Levine A.P., Segal A.W. (2013). What is wrong with granulocytes in inflammatory bowel diseases?. Dig. Dis..

[43] Marks D.J.B., Miyagi K., Rahman F.Z., Novelli M., Bloom S.L., Segal A.W. (2009). Inflammatory bowel disease in CGD reproduces the clinicopathological features of Crohn’s disease. Am. J. Gastroenterol..

[44] O’Morain C.A., Segal A.W., Walker D., Levi A.J. (1981). Abnormalities of neutrophil function do not cause the migration defect in Crohn’s disease. Gut.

[45] Sewell G.W., Marks D.J., Segal A.W. (2009). The immunopathogenesis of Crohn’s disease: A three-stage model. Curr. Opin. Immunol..

